# Longitudinal development of the anterior insula-nucleus accumbens white matter pathway through adolescence predicts risk taking in young adulthood

**DOI:** 10.1016/j.dcn.2026.101730

**Published:** 2026-04-24

**Authors:** Lauren R. Borchers, Chase Antonacci, Josiah K. Leong, Ian H. Gotlib

**Affiliations:** a Department of Psychology, Stanford University, 450 Jane Stanford Way, Stanford, CA 94305, USA; b Neurosciences Interdepartmental Program, Stanford University School of Medicine, 290 Jane Stanford Way, Stanford, CA 94305, USA; c Department of Psychological Science, University of Arkansas, 216 Memorial Hall, Fayetteville, AR 72701, USA; d Alice L. Walton School of Medicine, 1001 NE J Street, Bentonville, AR 72712, USA

**Keywords:** Anterior insula, Nucleus accumbens, Diffusion-weighted imaging, White matter, Risk taking, Adolescence

## Abstract

Adolescence is characterized by increases in risk-taking behaviors, including substance use, sexual activity, and impulsive decision-making. Neuroimaging research implicates the anterior insula (AIns) and nucleus accumbens (NAcc) in risk evaluation and reward motivation; however, little is known about how the structural pathway connecting these regions develops across adolescence or whether variation in its development predicts later risk-taking. In this study, we examined whether longitudinal changes in white-matter connectivity between the AIns and NAcc during adolescence predict risk-taking in early adulthood. A total of 196 youth (ages 9–20 years) contributed 486 diffusion-weighted imaging scans across four waves spanning six years. We reconstructed the AIns-NAcc tract using probabilistic tractography and extracted fractional anisotropy (FA) along the tract. At a fifth assessment (ages 19–23), 105 participants completed the Adolescent and Young Adult Health Questionnaire; exploratory factor analysis identified a risk-taking factor indexing substance use and sexual risk-taking. We used linear mixed-effects models to characterize developmental trajectories of FA and tested their associations with early adult risk-taking, adjusting for biological sex, early life stress, and behavioral sensitivity to reward and punishment. FA increased linearly across adolescence in both hemispheres (right: β=0.17, *p* < .001; left: β=0.10, *p* = .023). Critically, individuals exhibiting shallower increases in FA across adolescence in the right hemisphere reported greater engagement in risk-taking behaviors in early adulthood (ΔR^2^=4.0%, *p* = .024). These findings suggest that adolescence represents a sensitive period during which individual differences in maturation of the right AIns-NAcc pathway prospectively shape later risk-taking, highlighting the importance of longitudinally modeling structural connectivity in reward-related circuits.

## Highlights

• Anterior insula and nucleus accumbens are critical for risk and reward regulation.
• We modeled longitudinal trajectories of AIns-NAcc white-matter connectivity.
• Fractional anisotropy of the AIns-NAcc tract increases across adolescence.
• Right-hemisphere tract development predicts risk-taking behaviors in adulthood.
• Structural connectivity patterns advance our understanding of adolescent risk-taking.

## 1 Introduction

Adolescence is a critical period of development for neural circuits implicated in the processing and evaluation of risk and reward (C. F. Geier, 2013; Walker et al., 2017). Compared with adults, adolescents more frequently approach potentially rewarding stimuli (Galvan, 2010), seek novelty (Collado et al., 2014), and demonstrate poorer inhibitory control (Ferguson et al., 2021). Further, in typically developing adolescents behavioral sensitivity to reward follows an inverted U-shaped trajectory, increasing from childhood into adolescence and declining in early adulthood (Urošević et al., 2012). These characteristics have been posited to contribute to increased engagement in risky behaviors, including drunk driving (Arnett, 1990, Scott-Parker and Weston, 2017), risky sexual behaviors, and substance use (Poulton and Hester, 2019, Tapert and Eberson-Shumate, 2022), many of which peak during adolescence (Braams and van Duijvenvoorde, 2015, Willoughby et al., 2021).

In an effort to gain a more comprehensive understanding of the neural foundations of heightened reward-seeking, impulsivity, and risk-taking during this period, researchers have proposed that subcortical regions that support reward processing mature earlier than do prefrontal cortical regions involved in regulatory control (Galvan, 2013, Galvan et al., 2006; C. Geier and Luna, 2009). The nucleus accumbens (NAcc) is a central structure within this circuitry; it receives dopaminergic projections from the ventral tegmental area (Fitzgerald and Day, 2025, Ikemoto and Panksepp, 1999) and countervailing glutamatergic projections from the anterior insula (Ferenczi et al., 2016, Reynolds and Zahm, 2005). Functional neuroimaging studies have found that whereas NAcc activation tracks reward anticipation and approach behavior (Braams and van Duijvenvoorde, 2015, Casey et al., 2008), AIns activation is associated with the processing of risk, uncertainty, and potential losses (Preuschoff et al., 2008). Together, these findings have informed “imbalance” models of adolescent risk-taking, which implicate developmental asymmetries between regulatory and reward-related systems. These accounts are often formalized within dual-systems or imbalance frameworks (Casey et al., 2008, Steinberg, 2010), as well as in triadic models that include salience-processing regions such as the insula alongside reward and control systems (Ernst, 2014). Collectively, these models predict that heightened adolescent risk-taking reflects asynchronous maturation of motivational and regulatory circuits, with developmental changes in connectivity among these systems shaping behavioral outcomes. It is important to note, however, that most empirical tests of these frameworks have relied on functional activation; therefore, it is not clear whether or how the structural maturation of reward pathways contributes to behavioral outcomes.

In this context, converging findings from diffusion-weighted imaging (DWI) studies indicate that white matter pathways supporting frontostriatal, limbic, and salience networks continue to mature into the third decade of life (Lebel and Beaulieu, 2011). Specifically, longitudinal investigations document age-related increases in fractional anisotropy (FA) and decreases in mean diffusivity (MD), reflecting progressive refinement of axonal organization, packing density, and myelination (Genc et al., 2023, Tamnes et al., 2018). These protracted developmental trajectories are particularly pronounced in association fibers that connect cortical and striatal regions, in contrast to earlier-maturing sensorimotor tracts.

Importantly, individual differences in white matter microstructure have been linked to variability in impulsivity, sensation seeking, and substance use during adolescence and young adulthood. For example, structural properties of frontostriatal and limbic tracts, including the uncinate fasciculus, cingulum bundle, and corticostriatal pathways, have been associated with reward sensitivity, externalizing behaviors, and risk-taking propensity (Olson et al., 2009, Peper et al., 2013, Simmonds et al., 2014). Further, several longitudinal studies have demonstrated that variability in white matter maturation predicts trajectories of impulsivity, substance use initiation, and socioemotional functioning across adolescence (Green et al., 2023, Jacobus et al., 2013, Squeglia and Gray, 2016). Notably, these associations are not limited to broad frontocortical pathways. In fact, microstructural variation within circuits that support salience detection and reward valuation has been linked to risky decision-making and substance use vulnerability (Kohno et al., 2017), suggesting that connectivity within motivational networks is particularly relevant for understanding engagement in real-world risk-taking behaviors. However, the longitudinal development of more circumscribed salience-to-reward projections, such as the AIns-NAcc tract, and its links to real-world risk taking remain poorly understood.

Comparative studies have identified monosynaptic glutamatergic projections from the AIns to the NAcc (Chikama and McFarland, 1997, Reynolds and Zahm, 2005); recent work in humans has delineated this tract using diffusion-weighted imaging (Leong et al., 2016, Leong et al., 2018, Leong et al., 2021, Tisdall et al., 2022). Structural coherence of the AIns-NAcc tract has been linked to risk-related behaviors; specifically, greater tract coherence has been associated with decreased preference for risky gambles in adults (Leong et al., 2016), delayed relapse in patients with stimulant use disorder (Tisdall et al., 2022), and stronger reward drive in children (Leong et al., 2021). Despite these findings, the developmental trajectory of this pathway has not yet been characterized, nor have researchers tested whether characteristics of this pathway in adolescence predict real-world risk-taking behaviors in early adulthood.

In the current study we leveraged four waves of diffusion-weighted imaging (DWI) data collected over six years across adolescence to quantify developmental changes in the AIns-NAcc tract and to test whether these trajectories predict engagement in risky behaviors in early adulthood. We hypothesized that: (i) fractional anisotropy (FA) in the AIns-NAcc tract will increase with age, consistent both with evidence that association fibers connecting cortical and striatal regions show protracted maturation across adolescence and early adulthood (e.g., Lebel and Beaulieu, 2011; Tamnes et al., 2018) and with developmental patterns observed in comparable frontostriatal and salience-related pathways. Second, we hypothesized (ii) a shallower developmental increase in AIns-NAcc tract coherence will predict greater engagement in risky behaviors in early adulthood, consistent with imbalance models emphasizing salience and regulatory inputs to reward circuitry (Casey et al., 2008) and with findings of prior research linking stronger AIns-NAcc connectivity to reduced risky decision making (Leong et al., 2016). Finally, we examined whether the development of AIns-NAcc tract coherence predicts risk-taking above and beyond the variance contributed by the severity of early life stress (ELS) and behavioral sensitivity to reward and punishment, given their established associations with risk-taking behaviors (Hadaschik et al., 2025, Scott-Parker and Weston, 2017).

## 2 Methods

### 2.1 Participants and study design

**Fig. 1:**
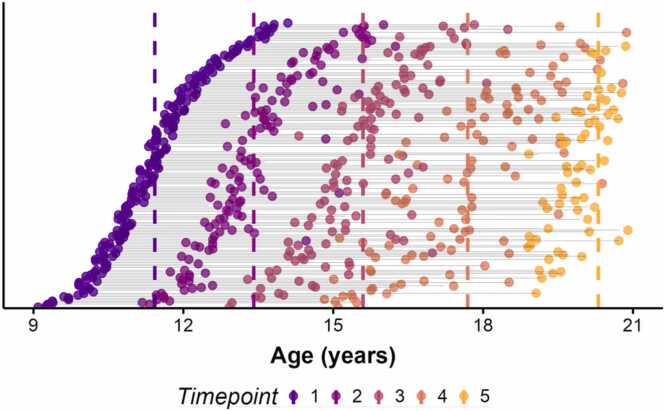
**Age Across Timepoints. Note.** Each row is a different participant. Dashed colored lines represent the mean age at the corresponding timepoint. The top section shows participants with two usable scans, followed by participants with three and four usable scans. Timepoint 5 was the risk-behavior assessment.

196 participants were included in the current study. Boys and girls from the San Francisco Bay Area were recruited as part of a larger study examining the psychobiological effects of early life stress (ELS) across puberty (Chahal et al., 2021, Ho et al., 2020, King et al., 2019). Prior publications from this cohort have examined associations among ELS, pubertal development, white matter microstructure, and internalizing and externalizing symptoms, but none has characterized the longitudinal development of the AIns-NAcc tract or its relation to later risk-taking behaviors. The current study includes up to five assessments per participant (*M*=3.34, *SD*=1.26; 1 session: *n* = 16; 2 sessions: *n* = 40; 3 sessions: *n* = 46; 4 sessions, *n* = 50; 5 sessions: *n* = 44; Fig. 1). The first four assessments included diffusion-weighted imaging and were conducted approximately two years apart beginning at ages 9–13 years; the fifth assessment included only a behavioral measure of risk-taking. The majority of participants had 2 or more usable DWI scans (M=2.46, *SD*=0.98; 1 scan: *n* = 32; 2 scans: *n* = 72; 3 scans: *n* = 58; 4 scans: *n* = 34). All participants with at least one usable DWI scan (N = 196) were included in modeling developmental trajectories of AIns-NAcc tract coherence. 105 participants completed the behavioral outcome assessment at the final timepoint and were included in analyses predicting risk-taking behaviors in early adulthood. All study procedures were conducted in compliance with relevant laws and institutional guidelines and were approved by the Stanford University Institutional Review Board (protocol 27671, approved 03/2025). Privacy rights of human subjects were observed. When participants were younger than 18 years of age, we obtained parental consent and participant assent; for assessments conducted when participants were 18 years of age or older, participants gave informed consent.

### 2.2 Diffusion-weighted imaging (DWI; Timepoints 1–4)

#### 2.2.1 DWI acquisition

MRI scans were acquired at the Center for Cognitive and Neurobiological Imaging (CNI) at Stanford University using a 3 T Discovery MR750 (GE Medical Systems, Milwaukee, WI) and a 32-channel head coil (Nova Medical). Diffusion-weighted images were collected using a single-shot spin-echo echo-planar imaging (EPI) sequence with anterior-posterior phase encoding (TR/TE=8500/93.5 ms; 64 axial slices; 2 mm isotropic voxels; FoV=260 mm; matrix=128 ×128; 60 b=2000 diffusion-weighted directions; 6 b=0 acquisitions; scan time≈9.5 min). No prospective motion correction was applied during acquisition. A T1-weighted anatomical scan was acquired for co-registration of the DWI data and to specify regions-of-interest (ROIs) for tractography (SPGR sequence; TR/TE/TI=6.24/2.34/450 ms; flip angle=12°; sagittal slices; 0.9 mm isotropic voxels).

#### 2.2.2 DWI preprocessing and tractography

Anatomical landmarks were manually placed at the anterior and posterior commissures (AC-PC), and the midsagittal plane to guide a rigid-body transformation of the T1-weighted images to AC-PC aligned space. Participant motion in the diffusion-weighted images was corrected by registering to the mean of the six motion-corrected non-diffusion-weighted (b=0) images. The mean of the non-diffusion-weighted images was aligned to the T1 image in AC-PC space using a rigid-body transformation. All raw diffusion images were resampled to 2 mm isotropic voxels by combining motion correction and anatomical alignment into one transformation and resampling the data using a seventh-order b-spline algorithm. All preprocessing steps were performed using the open-source mrDiffusion package in MATLAB (www.github.com/vistalab/vistasoft).

To define anatomical ROIs, each participant’s AC-PC aligned T1-weighted image was processed using FreeSurfer (version 6.0.1; Fischl, 2012). AIns ROIs were derived from the Destrieux 2009 atlas by combining the anterior insula and short gyrus parcellations (Destrieux et al., 2010, Leong et al., 2016). NAcc ROIs were identified from probabilistic subcortical tissue classification based on a manually labeled training set (Desikan et al., 2006). A binary white-matter mask was formed using the white/gray matter boundary defined by FreeSurfer. The ROIs were visually inspected for each participant to ensure they covered the relevant brain regions, and were additionally processed to fill holes in the ROI, remove satellite voxels, and dilated 1 voxel. This additional processing of the ROIs helped to prevent circular tracking within the ROI, and ensured that fibers successfully exited the ROI.

Fiber tracking between the AIns and NAcc ROIs was performed using constrained spherical deconvolution-based probabilistic tracking, as implemented in MRtrix software (version 0.2.12; Tournier et al., 2007). The method has been previously described (Leong et al., 2016, Leong et al., 2018) and open-source MATLAB code is available publicly (https://github.com/josiahkl/spantracts). The maximum number of harmonics was set to 6 (L_max_=6). Fiber pathways were generated by randomly seeding a voxel in a starting ROI and tracking until the fiber reached the ending ROI to ensure symmetrical fiber tracking (maximum number of fibers=5000, FA cutoff=0.1, curvature=60 °). Fibers leaving the white-matter mask were discarded.

We reduced the tractography results to a core fiber bundle by eliminating outliers and streamlines with indirect/looping trajectories inconsistent with a direct path between the AIns and NAcc ROIs. Specifically, we first removed fibers longer or shorter than 2 standard deviations from the mean fiber length. Next, we removed fibers greater than 3 standard deviations away from the mean spatial position of the core fiber (Mahalanobis distance). Finally, we removed fibers that took indirect routes between ROIs (e.g., fibers that started projecting in one direction but then looped backward).

#### 2.2.3 Structural coherence of the AIns-NAcc tract

**Fig. 2:**
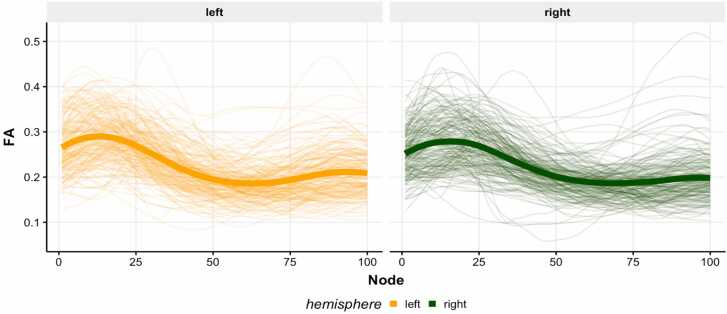
**Tract Profiles for the Left and Right Hemisphere. Note.** Node 1 corresponds to the start of the tract near the AIns, and node 100 corresponds to the end of the tract near the NAcc. Each thin line represents an individual participant’s average FA profile across all available timepoints; the thick line represents the mean profile for each hemisphere.

After characterizing the AIns-NAcc tract in all participants, we quantified the structural coherence of the tract by extracting diffusion metrics such as mean fractional anisotropy (FA). To assess FA along the spatial trajectory of the tract, we spatially normalized the tract by sampling 100 evenly-spaced cross-sectional nodes along the length of the tract from the start ROI to the end ROI (Ho et al., 2017, Yeatman et al., 2012). The mean FA in each node of the tract was then calculated by averaging FA from all fibers within a node, weighted by the Mahalanobis distance of a fiber from the node’s core fiber. We winsorized FA within each timepoint to 3 SDs above or below the mean to minimize the impact of extreme cases on developmental trajectories. We then calculated structural coherence of the left and right tracts by averaging FA across the full length of the tract (Fig. 2). This procedure generated a single mean FA value for the tract in each hemisphere of every participant and provided the estimate of structural tract coherence.

**Fig. 3:**
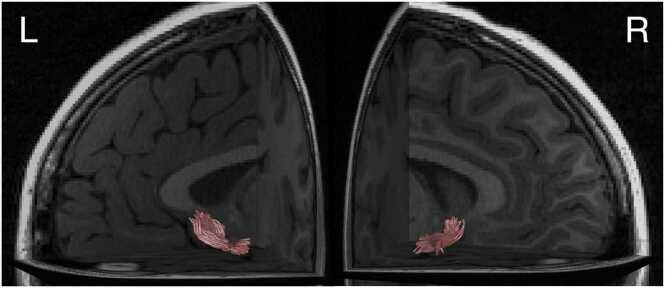
**Anterior Insula-Nucleus Accumbens Tract**. **Note.** Tract visualized in the left and right hemisphere in a representative participant.

To facilitate the anatomical localization of any tract-level effects, we conducted exploratory segment-level analyses, consistent with tract-profile work showing that diffusion properties can vary systematically along a tract due to fiber geometry, crossing fibers, and partial volume effects (Yeatman et al., 2012). Following Ho et al. (2017), we divided the tract into thirds to better localize the between-subjects associations with tract diffusion metrics while limiting multiple comparisons (first 25%: nodes 1–25 near the AIns and crossing the extreme and external capsules; middle 50%: nodes 26–75 traversing the sub-caudate white matter; final 25%: nodes 76–100 near the NAcc and crossing the internal capsule). These follow-up analyses were not associated with *a priori* hypotheses about specific tract segments. The AIns-NAcc tract in a representative participant is presented in Fig. 3. Using the same procedure, we also extracted axial diffusivity (AD) and radial diffusivity (RD) across the full tract to determine which underlying diffusion properties were driving the FA associations. We also extracted mean diffusivity (MD) as a complementary index of overall diffusivity magnitude. Finally, as a sensitivity analysis, we examined the corticospinal tract (CST) as a control tract using the same preprocessing and tract-profile procedures described above. We extracted mean FA across the full length of the CST at each available imaging timepoint and modeled its developmental trajectory in parallel with the AIns-NAcc tract.

### 2.3 Outcome measure: risk-taking (Timepoint 5)

Participants completed the Adolescent and Young Adult Health Questionnaire (AYAHQ; Minnesota Department of Health, 2023). The AYAHQ includes 29 items indexing physical, mental, and social well-being, covering constructs such as general health, safety, nutrition, sexual health, and substance use (a complete list of AYAHQ items is presented in the Supplement). Items such as sex and sexting were indexed across the lifetime using the options of ‘yes,’ ‘sometimes,’ and ‘no.’ Substance-related items including alcohol, nicotine, cannabis, and drug use were measured over the past year using ordinal response options: ‘never,’ ‘once or twice,’ ‘monthly,’ and ‘weekly.’ To reduce dimensionality, we conducted an exploratory factor analysis (EFA) of the AYAHQ items using the “fa” function from the *psych* package (Revelle, 2007), specifying minimum residual extraction with varimax rotation and pairwise correlations. Eight factors with eigenvalues> 1 were retained, consistent with inspection of the scree plot (see Supplemental Figure 1S). Regression-based factor scores were computed for each participant. Given our interest in risk-taking behaviors, we selected the factor characterized by high loadings (≥0.4) on alcohol, tobacco, marijuana, and other drug use, as well as sex and sexting, as our primary outcome measure (see Supplemental Figure 2S for full loadings and factor structure).

### 2.4 Covariates

#### 2.4.1 Early life stress (Timepoint 1)

To measure ELS, trained interviewers administered the Traumatic Events Screening Inventory for Children (TESI-C) to all participants at baseline. We obtained information about 30 different stressors commonly experienced in childhood in addition to an open-ended question where participants could report on any stressor that was not captured in the interview. Three or four coders blind to the interview determined the objective severity of each event using a modified version of the UCLA Life Stress Interview coding system (Rudolph et al., 2000). The agreement among coders was high (intraclass correlation coefficient ICC=0.99; King et al., 2019). An ELS severity score was calculated based on sum of the maximum score of each type of stressor endorsed in order to not overweight the scores of participants who reported many events. More information about how the TESI-C was scored can be found in King et al., (2019). This summed severity score was entered as a covariate in predictive models of risk-taking behaviors.

#### 2.4.2 Behavioral sensitivity to reward and punishment (Timepoints 1–4)

To assess behavioral sensitivity to reward, a documented predictor of adolescent risk-taking behaviors, we administered the Sensitivity to Punishment and Sensitivity to Reward Questionnaire for Children (SPSRQ-C; O’Connor et al., 2004) at timepoints 1–4. This frequently used 29-item questionnaire has good psychometric properties. Items index social sensitivity to reward and punishment (e.g., reward: “I like to do things that bring me immediate rewards,” “I want to have excitement and new feelings;” e.g., punishment: “In new tasks, I worry about failure,” “I often don’t do new things because I am afraid of being embarrassed”). Participants responded using a 5-point scale (“Strongly Disagree” to “Strongly Agree”). Because SPSRQ-C subscale scores demonstrated strong temporal reliability in our sample (ICC_3_k = 0.80–0.83), consistent with prior evidence of relative trait-like stability across adolescence (Conner et al., 2018), we averaged each subscale across timepoints 1–4 to index stable individual differences in reward and punishment sensitivity. These averaged scores were entered as covariates in predictive models of risk-taking behaviors assessed at timepoint 5.

### 2.5 Statistical analyses

All analyses were conducted in *R*. We compared participants who completed the final assessment to those who did not in order to examine whether completers differed from non-completers on demographic or relevant baseline variables. We examined both linear and quadratic changes in the AIns-NAcc tract across childhood and adolescence. Longitudinal trajectories were modeled using linear mixed-effects models with random intercepts and random slopes for age, nested within participant. Trajectories from the best fitting model were entered into linear regression models predicting risk-taking behaviors, one for each hemisphere. When FA trajectories significantly predicted scores on the risk-taking factor, we conducted supplemental exploratory analyses examining associations with individual risk-taking behaviors contributing to the factor (e.g., specific substance use and sexual risk items). Biological sex, ELS severity, and average SPSRQ-C reward and punishment sensitivity were included as covariates (see Supplementary Figure 3S for intercorrelations among primary behavioral and neural variables). When FA trajectories predicted risk taking, we conducted follow-up analyses to determine which portion of the tract was driving the prediction in that hemisphere (i.e., first 25%, middle 25%, final 25%). In addition, for any significant associations between FA and the risk-taking factor, we examined AD and RD to aid interpretation of the FA findings and clarify which diffusion components were driving the effect. We also examined mean MD as a complementary metric indexing the overall magnitude of diffusivity to determine whether associations extended beyond FA. Finally, to assess specificity, we modeled FA trajectories in the CST as a control tract and tested whether trajectories of CST FA predicted risk-taking behavior. Because we limited primary hypothesis testing to two *a priori* hemisphere-specific predictive models (left and right AIns-NAcc FA trajectories predicting risk-taking), with segment and diffusivity analyses treated as exploratory, we did not apply formal multiple-comparisons correction.

## 3 Results

### 3.1 Sample characteristics

Demographics characteristics of the sample are presented in Table 1. Participants who completed the final session (*n* = 105) did not differ from participants who did not complete the final session (*n* = 91) with respect to biological sex (χ^2^=0.00, *p* = 1.00), race (χ^2^=4.25, *p* = .514), age at baseline (*t* = 0.91, *p* = .365), behavioral sensitivity to reward (*t* = 0.80, *p* = .427) or punishment (*t* = 0.11, *p* = .910), exposure to ELS (*t* = -1.76, *p* = .081), or FA of the AIns-NAcc tract in the left (*t* = 0.04, *p* = .971) or right (*t* = -1.89, *p* = .061) hemisphere.

**Table 1:** Sample Characteristics.

**AIns-Nacc Trajectory Modeling (N = 196)**	
**Variable**	***M*****(*****SD*****);*****n*****(%)**
**Sex**	117 female (59.7%)
**Race**	
Asian/Asian American	22 (11.2%)
Biracial	41 (20.9%)
Black/African American	15 (7.7%)
Hispanic/Latin-X	15 (7.7%)
Other Race	12 (6.1%)
White	91 (46.4%)
Missing data	1 (0.5%)
**N contributing scans at Time 1**	138
Age Time 1	11.43 (1.06)
NAcc-AIns FA Time 1	Left: 0.22 (0.03); Right: 0.21 (0.04)
**N contributing scans at Time 2**	136
Age Time 2	13.41 (1.17)
NAcc-AIns FA Time 2	Left: 0.22 (0.03); Right: 0.22 (0.03)
**N contributing scans at Time 3**	113
Age Time 3	15.60 (1.12)
NAcc-AIns FA Time 3	Left: 0.23 (0.04); Right: 0.23 (0.04)
**N contributing scans at Time 4**	99
Age Time 4	17.69 (1.46)
NAcc-AIns FA Time 4	Left: 0.23 (0.04); Right: 0.23 (0.04)
**Risk-Taking Predictive Modeling (N = 105)**	
Age Time 5	20.31 (1.00)
Sex	63 female (60.0%)
Risk Factor Score (Time 5)	3.83 (2.64)
Early Life Stress Severity (Time 1)	6.00 (4.50)
Reward Sensitivity Average (Time 1–4)	2.87 (0.53)
Punishment Sensitivity Average (Time 1–4)	2.81 (0.63)

**Note.** Sample characteristics table. AIns = anterior insula; NAcc = nucleus accumbens, FA = fractional anisotropy.

### 3.2 Developmental trajectories of AIns-NAcc white matter connectivity

Across the entire sample of participants with usable DWI data for at least one timepoint, we found that including the quadradic term did not significantly improve model fit for either the left (χ²(1)= 0.72, *p* = .396) or the right (χ²(1)= 2.93, *p* = .087) hemisphere; therefore, in all subsequent analyses we used trajectories extracted from linear mixed effects models. We found significant linear age-related increases in the left (*b*=0.0014, *SE*=0.0006, *t* = 2.30, *p* = .023; standardized β=0.10) and right (*b*=0.0025, *SE*=0.0007, *t* = 3.69, *p* < .001; standardized β=0.17) AIns-NAcc tracts (see Supplemental Figure 4S; Table 1S). These models included a random intercept and slope for age, allowing for individual variation across participants. We found significant age-related changes in the middle portion of the left (*b*=0.0023, *SE*=0.0007, *t* = 3.24, *p* = .001; standardized β=0.14) and right (*b*=0.0033, *SE*=0.0008, *t* = 4.06, *p* < .001; standardized β=0.19) AIns-NAcc tracts, as well as in the last quarter of the tract in the right (*b*=0.0029, *SE*=0.0010, *t* = 2.86, *p* = .005; standardized β=0.13), but not in the left (*p* = .162), hemisphere; there were no age-related changes in the first quarter of the tract in either the left (*p* = .633) or the right (*p* = .617) hemisphere.

### 3.3 Factor analysis of items assessing risk-taking behavior

One item from the AYAHQ had 0 endorsement (gang membership) and was excluded from analysis. An EFA conducted on the remaining AYAHQ items yielded eight factors with eigenvalues > 1 that explained 55% of the variance in AYAHQ scores (see Supplemental Figure 1S for the Scree plot). All factors and a heatmap plotting the item loadings are presented in the Supplement (Fig. 2S). Given our interest in risk-taking behaviors, we focused on the factor that most clearly indexed risk taking, which included the following items that loaded > 0.40: sex, sexting, alcohol use, nicotine use, cannabis use, and other drug use. This factor explained 10% of the variance in the AYAHQ. Supplemental analyses examining the specificity of our findings to risk-taking behaviors are presented in the Supplement (Figure 5S).

### 3.4 Trajectories of AIns-Nacc tract connectivity predict risk-taking behaviors

**Fig. 4:**
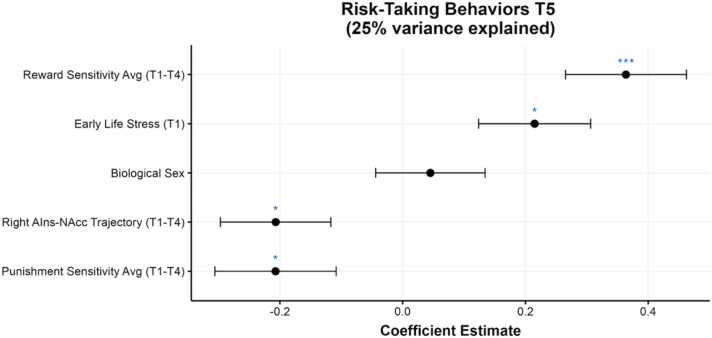
**Predictors of Risk-Taking Behaviors in Adulthood. Note.** Plotting results of regression model.

Our base model including biological sex, ELS, and average sensitivity to reward and punishment across timepoints 1–4 explained 20.6% of the variance in risk-taking behaviors assessed in early adulthood, with ELS (*b*=0.138, *SE*=.054, *t* = 2.548, *p* = .012), sensitivity to reward (*b*=1.950, *SE*=0.499, *t* = 3.906, *p* < .001), and sensitivity to punishment (*b*=-1.051, *SE*=0.415, *t* = -2.531, *p* = .013) each contributing unique variance (Fig. 4). We found that trajectories of AIns-NAcc tract FA in the right (*R*^2^Δ=4.0%, *p* = .024), but not in the left (*R*^2^Δ=1.7%, *p* = .147), hemisphere predicted unique variance in risk-taking scores in young adulthood, with shallower (or decreasing) trajectories of FA predicting more risk-taking behaviors (Fig. 4). More specifically, we found that shallower trajectories of right AIns-NAcc FA in the middle portion (*R*^*2*^Δ=3.5%, *p* = .036) and in the last quarter (*R*^*2*^Δ=5.7%, *p* = .007) of the tract near the NAcc juncture predicted more risk-taking behaviors in early adulthood; FA in the first quarter of the tract did not predict subsequent risk-taking behaviors (*R*^2^Δ=0.12%, *p* = .703).

### 3.5 Axial, radial, and mean diffusivity

To further characterize the microstructural properties associated with risk-taking behaviors, we examined trajectories of AD, RD, and MD in the right hemisphere of the AIns-NAcc tract (see Figure 6S for associations between FA, AD, MD, and RD across timepoints). We found, overall, that trajectories of AD (*r* = 0.261, *p* = .007) and MD (*r* = 0.199, *p* = .041), but not RD (*r* = 0.092, *p* = .350), were associated with the risk-taking factor. More specifically, we found that within the middle 50% of the tract, AD (*r* = 0.204, *p* = .037), but not MD (*r* = 0.166, *p* = .090) or RD (*r* = 0.068, *p* = .488) predicted risk-taking; similarly, in the final 25% of the tract, only AD (*r* = 0.249, *p* = .010) significantly predicted risk taking (MD: *r* = -0.014, *p* = .886; RD: *r* = 0.182, *p* = .063).

### 3.6 Specific risk-taking behaviors

Pearson correlations indicated that the trajectory of the AIns-NAcc tract in the right hemisphere predicted specific risk-taking behaviors (see Figure 5S). Specifically, whereas shallower trajectories of FA of middle portion of the right AIns-NAcc tract was related to more alcohol, marijuana, and other drug use, shallower FA of the last quarter of the AIns-NAcc tract was related to all risk-taking behaviors except alcohol and other drug use; FA in the first quarter of the tract was not related to any individual risk-taking behaviors.

### 3.7 Control tract analyses

Finally, to assess whether the effects of structural coherence on risk-taking behaviors were specific to the AIns-NAcc tract, we modeled the corticospinal tract (CST) as a control tract. In contrast to findings obtained for the AIns-NAcc tract, we found significant age-related *decreases* in FA both in the right (*b*=-0.0032, *SE*=0.0006, *t* = -5.16, *p* < .001; standardized β=-0.23) and the left (*b*=-0.0020, *SE*=0.0006, *t* = -3.43, *p* = .001; standardized β=-0.15) CST. Importantly, however, neither FA in the right (*p* = .669) nor in the left (*p* = .257) CST predicted risk-taking behaviors, suggesting that the observed effects are specific to coherence of the AIns-NAcc tract. Moreover, neither bivariate association between the risk-taking factor and the right and the left CST were significant (right: *r* = -0.024, *p* = .812; left: *r* = -0.108, *p* = .277).

## 4 Discussion

In this study, we examined the development of the AIns-NAcc white matter pathway across adolescence and tested whether individual differences in this maturation predict risk-taking behaviors in early adulthood. Across four waves spanning six years, we found that fractional anisotropy (FA) in the AIns-NAcc tract increased linearly from late childhood through early adulthood. Importantly, youth characterized by shallower or slower increases in the development of the AIns-NAcc tract, particularly in the right hemisphere, engaged in more risk-taking behaviors in early adulthood. These associations were driven by axial diffusivity (AD), not by radial diffusivity (RD), suggesting that axonal properties rather than myelin alterations underlie these individual differences. Finally, these effects were specific to the AIns-NAcc pathway and were not evident in a control tract. This is the first study to demonstrate that structural changes in this corticostriatal pathway across adolescence predict risk-taking behaviors in early adulthood.

The finding that FA increased in both hemispheres across adolescence is consistent with the broader literature documenting protracted white matter maturation during this period. Developmental neuroimaging studies consistently show increases in FA well into the third decade of life, reflecting age-related changes in axonal coherence, packing density, and myelination, particularly within frontostriatal and limbic pathways (Asato et al., 2010, Lebel and Beaulieu, 2011). The AIns-NAcc tract, which carries glutamatergic projections from the insula to the ventral striatum, appears to follow this general pattern of linear growth. This trajectory likely reflects the prolonged refinement of circuits supporting affective decision-making and interoceptive signaling, processes that reach functional maturity in early adulthood. Although many white matter tracts are characterized by nonlinear, decelerating increases in FA across adolescence, particularly in studies spanning childhood into the third decade of life (Lebel et al., 2012, Lebel and Beaulieu, 2011), our data best fit a linear model over the age range sampled here. Nonlinear components may have been modest within this developmental window, and detecting subtle curvature may require larger samples and/or denser assessments. Alternatively, association fibers that link salience and reward circuitry may follow a more gradual, extended maturation profile relative to earlier-maturing projection tracts. However, the presence of significant change in both hemispheres highlights the fact that the AIns-NAcc pathway undergoes meaningful developmental tuning across adolescence. Further, the somewhat larger developmental slope in the right hemisphere is consistent with known lateralization in insular structure and function (Duerden et al., 2013); the right AIns has frequently been linked to salience detection, inhibitory control, and interoceptive awareness (Cai and Ryali, 2014, Ghahremani et al., 2015). These asymmetries may help to explain why only right-hemisphere development predicted behavioral outcomes in this study.

A key contribution of our study is the demonstration that individual differences in the maturation of the AIns-NAcc tract through adolescence are meaningfully associated with subsequent risk-taking behaviors. Youth who had shallower increases in FA across adolescence engaged in more sexual risk-taking, substance use, and other health-risk behaviors in young adulthood. These findings suggest that slower or less robust strengthening of this pathway limits the extent to which insula-based information about risk, uncertainty, or aversive outcomes modulates NAcc-driven reward pursuit. This interpretation is consistent with findings from studies showing that whereas AIns activation tracks risk prediction errors and signals potential losses (e.g., Preuschoff et al., 2008), NAcc activation tracks reward anticipation and approach behaviors (e.g., Galvan et al., 2006). Considered together, these findings suggest that a structurally weaker or less mature AIns-NAcc pathway reduces the efficiency with which insular signals temper or contextualize striatal reward responses. Thus, adolescents with slower maturation of this tract might rely more heavily on reward-driven impulses and less on internally generated indicators of uncertainty or negative outcomes, increasing their likelihood of engaging in risky behaviors.

The specificity of effects to AD rather than RD is also noteworthy. Because AD is often interpreted as indexing axonal integrity or organizational coherence, and RD is related more closely to myelination processes, our findings suggest that individual differences in risk taking are tied more strongly to axonal organization within the tract than to myelin changes per se (Kumar et al., 2012). This formulation is consistent with findings of longitudinal studies showing that axonal packing and orientation continue to increase during adolescence and predict cognitive and socioemotional development (Genc et al., 2023, Mah et al., 2017). It also raises the possibility that the developmental refinement of this tract is driven primarily by axonal reorganization rather than by myelination, although caution is warranted given the indirect nature of diffusion metrics.

It is noteworthy that developmental trajectories in the middle portion of the tract and in the segment near the NAcc were related most strongly to subsequent risk-taking behaviors. These anatomical findings may be due to the middle portion of the tract likely reflecting deep white matter where fibers are well aligned and less influenced by partial volume effects, while the terminal tract segment captures the region in which insular fibers converge into the ventral striatum. In fact, this convergence zone may be especially important for integrating interoceptive or aversive signals with reward valuation. Reduced maturation in this segment may weaken the ability of the insula to modulate striatal activity, shifting the balance toward reward-drive processes that strengthen adolescents’ propensity to engage in risky behaviors.

These findings contribute to theoretical models that posit an imbalance between reward- and control-related systems during adolescence (Casey et al., 2008, Steinberg, 2010). Prior work has focused primarily on functional activation, showing heightened NAcc sensitivity to rewards and declining insula engagement across adolescence. Our findings complement this literature by documenting a structural tract that unfolds over the same developmental window. Rather than viewing functional activation patterns in isolation, our findings indicate that the white matter connectivity that supports communication between these regions also undergoes a prolonged period of maturation that has consequences for real-world behavior. This structural perspective also adds nuance to “imbalance” theories: specifically, variability in the maturation of regulatory projections into reward circuits may be a critical determinant of engaging in risky behaviors. It is also consistent with studies showing that greater structural coherence of the AIns-NAcc tract is related to reduced gambling risk in adults and slower relapse in individuals with stimulant use disorder (Leong et al., 2016, Tisdall et al., 2022). Our longitudinal findings extend this work by showing that the developmental slope of this tract, not simply its cross-sectional strength, predicts engagement in risk-taking behaviors years later.

Finally, the associations we documented in this study remained significant after accounting for sex, ELS, and behavioral sensitivity to reward and punishment, indicating that the developmental trajectory of the AIns-NAcc tract explains unique variance in later behavior. The absence of predictive effects in the CST further supports anatomical specificity and reduces the likelihood that the results reflect general white matter development or global maturation effects. Interestingly, FA in the CST decreased slightly across this age range. Projection fibers such as the CST typically mature earlier than association pathways, often showing plateauing during mid-to-late adolescence and, in some samples, slight decreases (Lebel et al., 2019). The modest decrease observed in the current study is therefore consistent with prior developmental work and may reflect ongoing microstructural reorganization during late adolescence, including changes in fiber complexity or other maturational processes. Importantly, CST trajectories were not associated with risk-taking behavior, underscoring the specificity of the AIns-NAcc findings. In addition, the pattern of associations across behavior types offers a useful context: whereas decreases in FA in the middle segment were most strongly linked to substance use, decreases in the terminal segment predicted a broader range of behaviors, including risky sexual activities. These differences underscore the value of conducting node-based and segment-specific analyses to identify which portions of a tract are functionally relevant.

We should note three limitations of this study. First, because of attrition we had a relatively small sample of participants with risk-taking behavior and uneven numbers of DWI scans across participants; importantly, however, the mixed-effects models we conducted mitigate this concern by accommodating unbalanced data. Second, although the NAcc-AIns tract has been well described in nonhuman primates (Chikama et al., 1997), tractography cannot determine directionality or guarantee the presence of monosynaptic connections, thus limiting our ability to make definitive statements about the functional interactions between these two brain regions. Finally, risk-taking behaviors were assessed only at the final wave, limiting inferences about bidirectional influences between white matter development and behavior. Future research should examine how structural maturation of the AIns-NAcc tract is related to developmental changes in functional connectivity and/or task activation to yield an integrated understanding of precisely how this pathway shapes risk-seeking behaviors. Similarly, studies incorporating genetic, hormonal, and/or environmental moderators may help explain why some adolescents exhibit slower maturation than others.

Despite these limitations, this study is important in demonstrating that the AIns-NAcc white matter pathway continues to mature across adolescence and that individual differences in this trajectory predict engagement in risk-taking behaviors in early adulthood. Thus, we have highlighted a structural pathway through which developmental processes influence real-world behavior, underscoring the importance of considering long-term patterns of white matter maturation in understanding adolescents’ risk for engaging in maladaptive behaviors.

## CRediT authorship contribution statement

**Borchers Lauren R:** Writing – review & editing, Writing – original draft, Formal analysis, Data curation, Conceptualization. **Josiah K. Leong:** Writing – review & editing, Funding acquisition, Data curation, Conceptualization. **Chase Antonacci:** Writing – review & editing, Writing – original draft, Formal analysis. **Ian H. Gotlib:** Writing – review & editing, Funding acquisition.

## Funding

This research was funded by the 10.13039/100000025National Institute of Mental Health (grant R37MH101495 to IHG) and Arkansas Biosciences Institute (seed funding to JKL).

## Declaration of Competing Interest

The authors report no competing interests.

## Data Availability

Data will be made available on request.
