# Supplementary material for: Longitudinal development of the anterior insula-nucleus accumbens white matter pathway through adolescence predicts risk taking in young adulthood
Source: Dev Cogn Neurosci. 2026 Apr 24;79:101730. doi: 10.1016/j.dcn.2026.101730 (PMC13137198; doi:10.1016/j.dcn.2026.101730)
Supplement: Supplementary file 1 — Supplementary material [file mmc1.docx]

**Supplemental Information**

*Longitudinal Development of the Anterior Insula-Nucleus Accumbens Pathway Through Adolescence Predicts Risk Taking in Young Adulthood*

**Supplementary Methods**

- AYAHQ complete list of items

**Supplementary Results**

- Figure 1S: Exploratory Factor Analysis (EFA) Scree Plot
- Figure 2S: Heat Map of Factor Loadings from EFA of AYAHQ
- Figure 3S. Intercorrelations Among Primary Behavioral and Neural Variables
- Figure 4S: Developmental Trajectories of AIns-NAcc Tract Fractional Anisotropy (FA) Across Adolescence
- Table 1S: Linear Mixed Effects Model Results
- Figure 5S: Associations between Right AIns-NAcc FA and Specific Risk-Taking Behaviors
- Figure 6S: FA, MD, AD, and RD in the Right AIns-NAcc Tract.

**Supplementary Methods**

Adolescent and Young Adult Health Questionnaire (AYAHQ) Items

| **We ask every patient these questions about things that can affect your health and well-being. Some of the questions might not fit you. It is okay to leave some questions blank. Please answer these questions on your own, without help from your parent or friends, and be as honest as possible. Your answers are private.** | **Please Circle**  **your anwer** |
| --- | --- |
| 1. In general, are you happy with the way things are going for you? | Yes Sometimes No |
| 1. Do you wear a seat belt in a car/truck? | Yes Sometimes No |
| 1. Do you wear a helmet when you skateboard, bicycle, motorcycle, snowmobile or ATV? | Yes Sometimes No |
| 1. Do you get along with your family? | Yes Sometimes No |
| 1. Do you have at least one adult you can really talk to? | Yes Sometimes No |
| 1. Do you feel safe at home, at school and in your community? | Yes Sometimes No |
| 1. Do you get 60 minutes of physical activity most days of the week? | Yes Sometimes No |
| 1. Do you think you are about the right weight and height? | Yes Sometimes No |
| 1. Do you ever skip meals, use laxatives or diet pills, or throw up on purpose to lose weight or to control your weight? | Yes Sometimes No |
| 1. Have you missed more than 7 days of school in the last year? | Yes Sometimes No |
| 1. Are your grades worse than they used to be? | Yes Sometimes No |
| 1. Do you or anyone you live with have a gun or carry around a gun? | Yes Sometimes No |
| 1. Do you worry about money, a place to live, food or clothing? | Yes Sometimes No |
| 1. Have you ever run away from home? | Yes Sometimes No |
| 1. Have you ever been in a gang (now or in the past)? | Yes Sometimes No |
| **Your answers are private between you and your health care provider. We will only talk to your parent/guardian about this information if we have a serious concern about your health and safety. Before we talk to a parent/guardian, we will talk about it with you.** | **Please Circle**  **your answer** |
| 1. Do you ever hurt or cut yourself on purpose? | Yes Sometimes No |
| 1. Have you ever texted/sent or received a sexual message or picture? | Yes Sometimes No |
| 1. Have you ever had any kind of sex (with anyone of any gender)? | Yes Sometimes No |
| 1. Have you ever had an infection that is spread by having sex? (like herpes, gonorrhea, chlamydia, genital warts, pelvic inflammatory disease, HIV, syphilis) | Yes Sometimes No |
| 1. Have you ever traded sex or sexual activity for money, food, a place to live, or anything else? | Yes Sometimes No |
| 1. Are you, or do you wonder if you are gay, lesbian, bisexual, pansexual, asexual, or other? | Yes Sometimes No |
| 1. Are you, or do wonder if you are transgender, gender diverse, or a gender that is different from what you were called (boy or girl) at birth? | Yes Sometimes No |
| 1. Have you ever been physically, sexually, or emotionally abused or hurt by anyone? (such as kicked, hit, forced or tricked into having sex, touched in a way that made you feel uncomfortable, called worthless) | Yes Sometimes No |
| 1. Have you ever, in your whole life, tried to kill yourself? | Yes Sometimes No |
| 1. Have you had any stressful or scary events that still bother you? | Yes Sometimes No |

Questions about tobacco, alcohol, marijuana, and other drugs

| **In the PAST YEAR, how many times have you used:** | **Never** | **Once or twice** | **Monthly** | **Weekly** |
| --- | --- | --- | --- | --- |
| 1. Tobacco, e-cigarettes or vapes,   such as JUUL, suorin, blu, VUSE, or logic? |  |  |  |  |
| 1. Alcohol |  |  |  |  |
| 1. Marijuana |  |  |  |  |
| 1. Have you tried any other drugs for fun, curiosity or coping, such as prescription pills, drugs that you sniff or huff, salvia, K2, or other illegal drugs? |  |  |  |  |

**Note.** The AYAHQ was administered at Time 5 (early adulthood; ages 19-23 years).

**Supplementary Results**

**Figure 1S.** Exploratory Factor Analysis Scree Plot


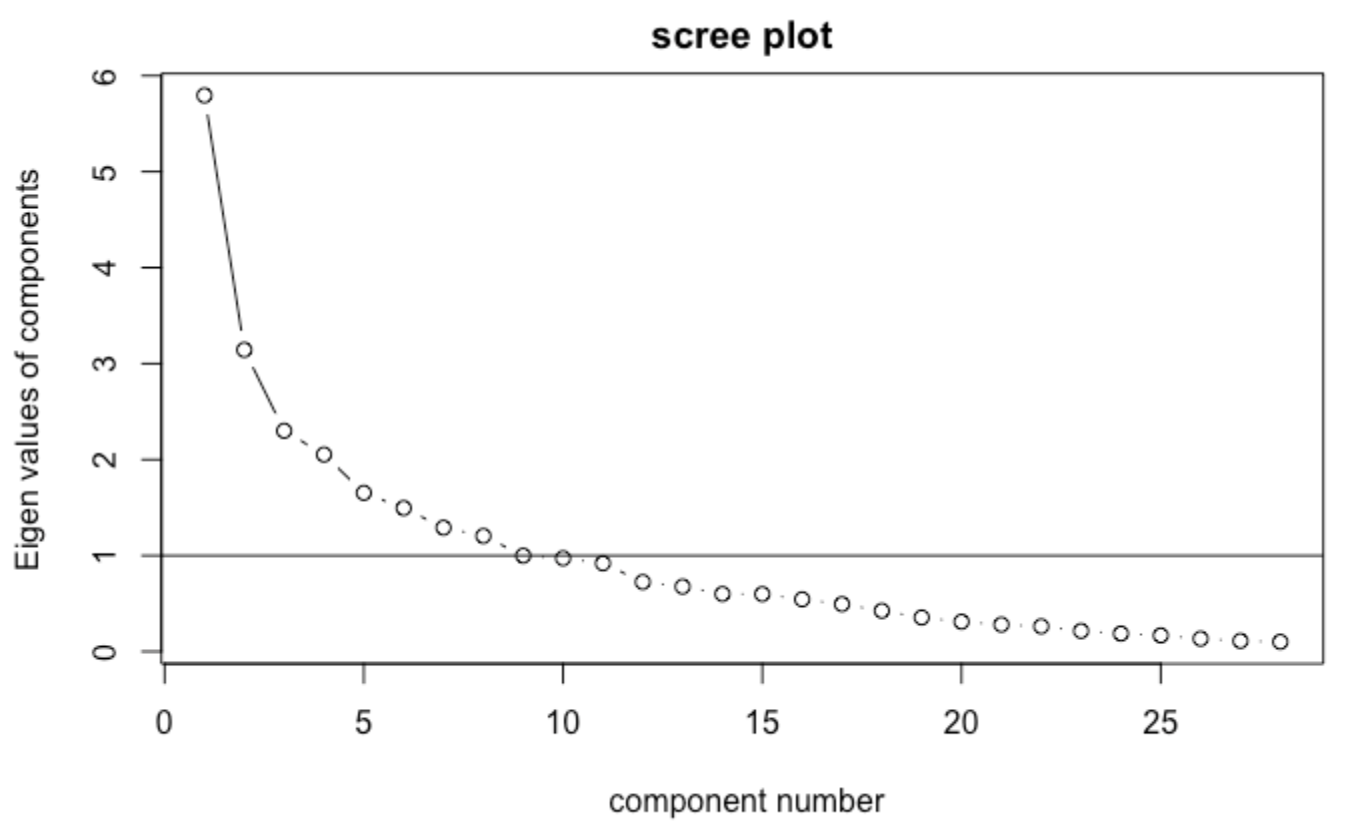


**Note.** Scree plot of factors derived from exploratory factor analysis of AYAHQ items conducted using the “fa” function of the *psych* package in R. The model specified eight factors using minimum residual extraction, varimax rotation, and pair wise correlations. Regression-based factor scores were computed for each participant.

**Figure 2S.** Heat Map of Factor Loadings from EFA of AYAHQ

**Note.** Factor Loadings from exploratory factor analysis of the Adolescent and Young Adult Health Questionnaire (AYAHQ) items. Factor 2 (loadings ≥ 0.4 for sexting, sex, alcohol, marijuana, tobacco, and other drug use) was extracted as the ‘risk-taking’ factor used as the outcome measure in primary analyses. STI = sextually transmitted infection; LGBT+ = gay, lesbian, bisexual, pansexual, asexual, or other; ED = eating disorder.


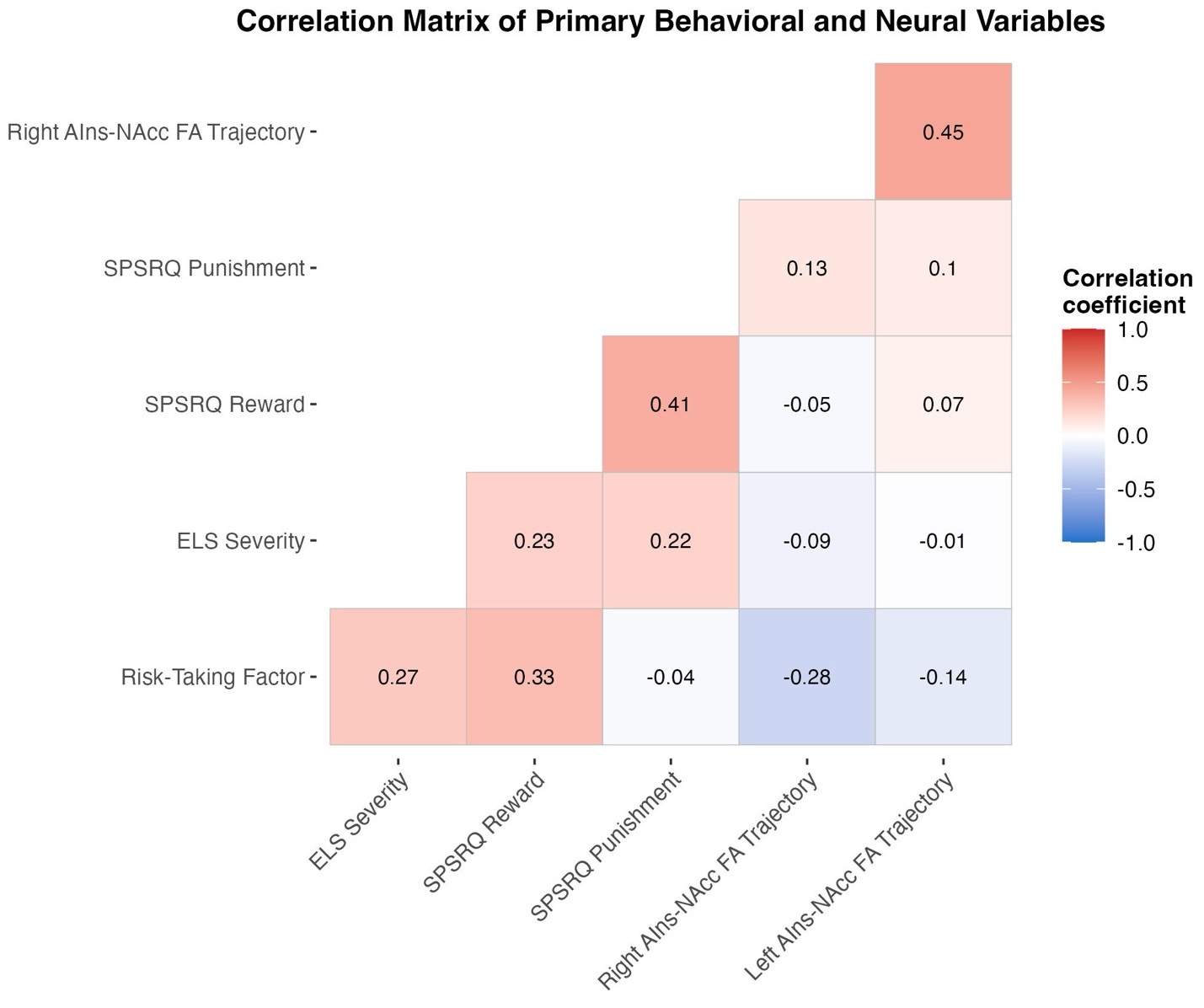
**Figure 3S.** Intercorrelations Among Primary Behavioral and Neural Variables

**Note.** Correlation matrix of primary behavioral and neural variables included in predictive models. Pearson correlation coefficients are shown for the risk-taking factor, early life stress (ELS) severity, SPSRQ-C reward and punishment sensitivity (averaged across Timepoints 1-4), and left and right AIns-NAcc FA trajectory parameters. Correlations were computed in the analytic sample included in predictive models (N=105). Color scale represents correlation magnitude (red=positive; blue=negative). SPSRQ-C=Sensitivity to Punishment and Sensitivity to Reward Questionnaire; AIns=anterior insula; NAcc=nucleus accumbens.

**
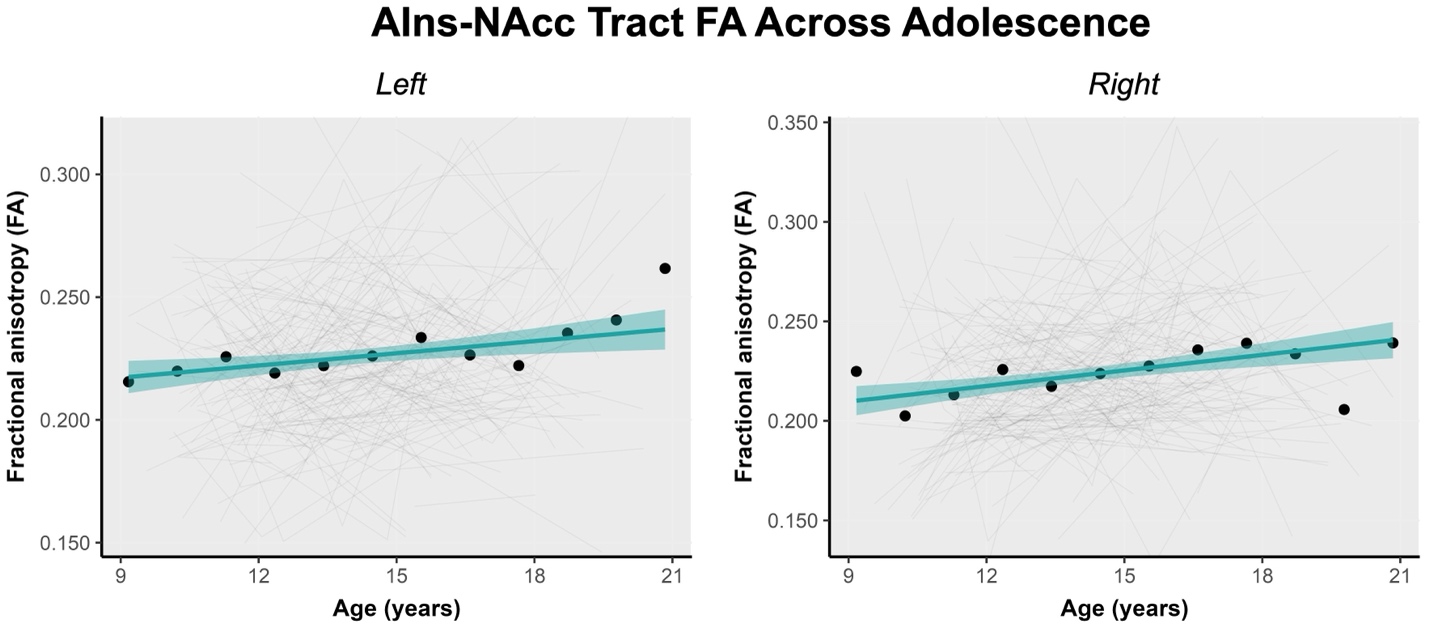
Figure 4S.** Developmental Trajectories of AIns-NAcc tract FA Across Adolescence

**Note.** Developmental trajectories of AIns-NAcc tract fractional anisotropy (FA) across adolescence. Spaghetti plots depict individual participant trajectories of FA across age for the left (left panel) and right (right panel) anterior insula-nucleus accumbens (AIns-NAcc) tract. Thin gray lines represent individual longitudinal observations. Black circles indicate mean FA within age bins. Solid aqua lines represent linear fits across age, with shaded regions denoting 95% confidence intervals. Note that there were relatively few DWI assessments at the oldest ages (~19+ years), so bin means in this range may be less stable. Both hemispheres demonstrate gradual increases in FA across adolescence.

**Table 1S.** Linear Mixed Effects Model Results

| **Left AIns-NAcc:** FA ~ age_centered + (1 + age_centered \| subject_id) | | | | | | | |
| --- | --- | --- | --- | --- | --- | --- | --- |
|  | ***Fixed Effects*** | | | | ***Random Effects*** | | |
| **Predictor** | ***β*** (unstandardized) | ***SE*** | ***t*** | ***p*** | **Variance** | ***SD*** | **Correlation** |
| Intercept | 0.2183 | 0.0033 | 66.94 | <.001 | - | - | - |
| Age_centered | 0.0014 | 0.0006 | 2.30 | .023 | - | - | - |
| Intercept | - | - | - | - | 0.000171 | 0.01309 | - |
| Age slope | - | - | - | - | 0.000006 | 0.00252 | -0.22 |
| Residual | - | - | - | - | 0.000793 | 0.02815 | - |
| **Right AIns-NAcc:** FA ~ age_centered + (1 + age_centered \| subject_id) | | | | | | | |
|  | ***Fixed Effects*** | | | | ***Random Effects*** | | |
| **Predictor** | ***β*** (unstandardized) | ***SE*** | ***t*** | ***p*** | **Variance** | ***SD*** | **Correlation** |
| Intercept | 0.2101 | 0.0037 | 56.45 | <.001 | - | - | - |
| Age_centered | 0.0025 | 0.0007 | 3.69 | <.001 | - | - | - |
| Intercept | - | - | - | - | 0.000100 | 0.00999 | - |
| Age slope | - | - | - | - | 0.000007 | 0.00273 | -0.42 |
| Residual | - | - | - | - | 0.001149 | 0.03389 | - |

**Note.** Linear mixed-effects models of left and right AIns-NAcc fractional anisotropy (FA) across adolescence. Models included age (centered at 9 years) as a fixed effect and random intercepts and random slopes for age nested within participant (subject_id). Fixed effects are reported as unstandardized coefficients (*β*), standard errors (*SE*), *t* values, and *p* values. Random effects are reported as variance components, standard deviations (*SD*), and correlations between random intercepts and slopes. Models were estimated using restricted maximum likelihood (REML), and degrees of freedom were calculated using the Satterthwaite approximation. N observations = 486; N participants = 196.

**Figure 5S.** Associations between Right AIns-NAcc FA and Specific Risk-Taking Behaviors
**
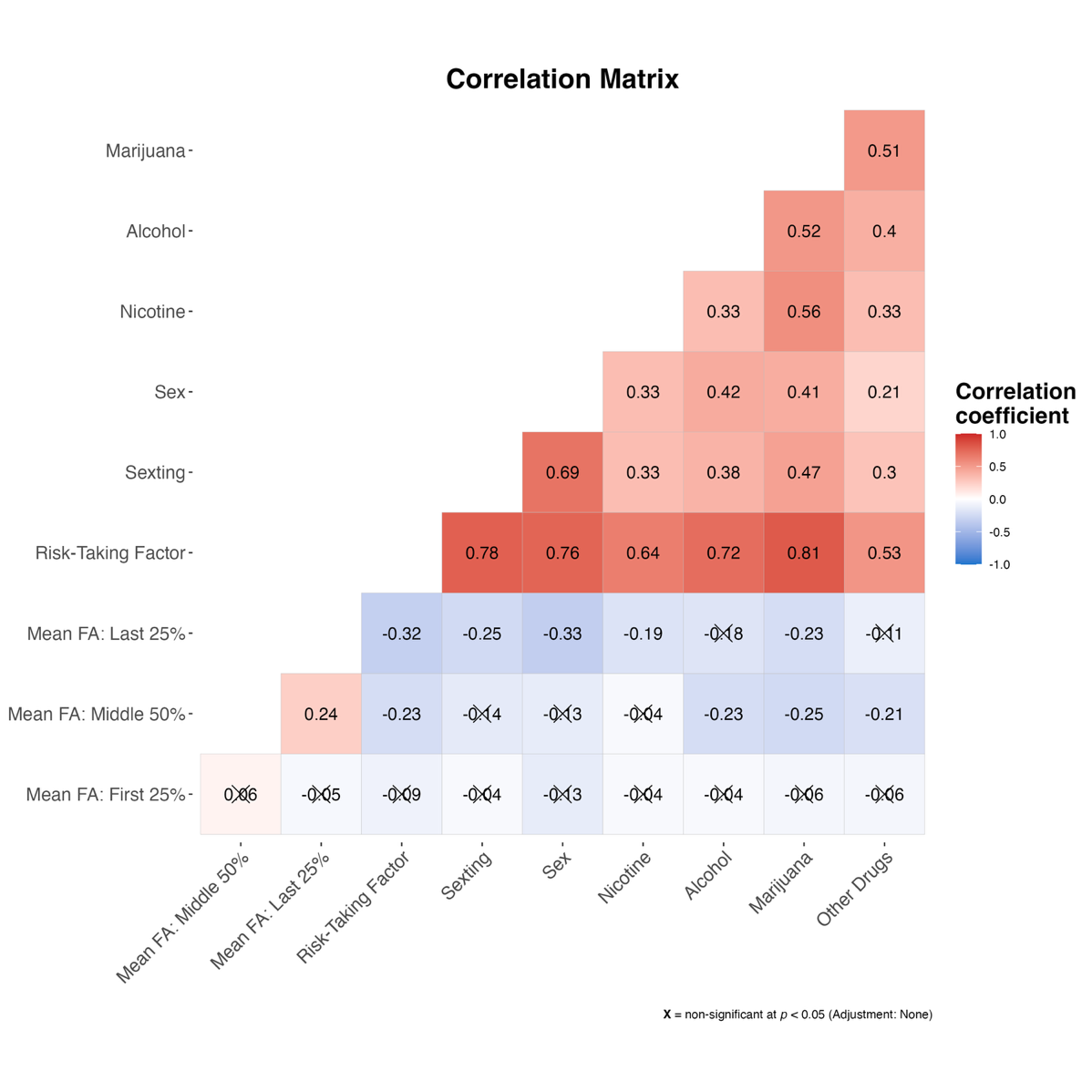
**

**Note.** Red = positive association, blue = negative association, x indicates nonsignificant association. First / Middle / Last = first 25% of the AIns-NAcc tract, middle 50% of the AIns-NAcc tract, and last 25% of the AIns-NAcc tract. FA = fractional anisotropy; AIns = anterior insula; NAcc = nucleus accumbens.


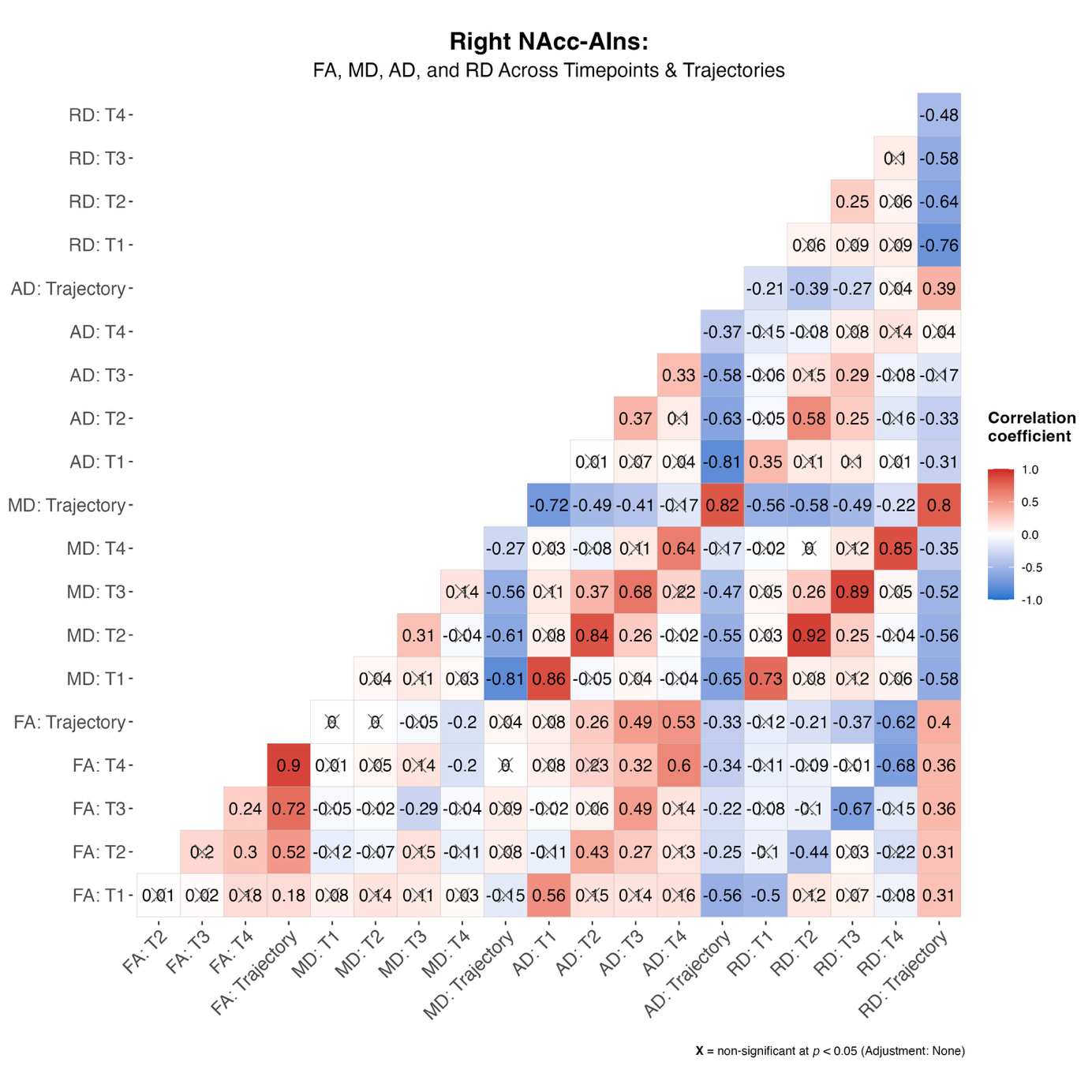
**Figure 6S.** FA, MD, AD, and RD in the Right Ains-NAcc Tract.

**Note.** Red = positive association, blue = negative association, x indicates nonsignificant association. FA = fractional anisotropy; MD = mean diffusivity; AD = axial diffusivity; RD = radial diffusivity; T1-T4 = timepoints 1-4.
